# Evaluation of Patients With Positive Patch Test Reactions to Rubber Additives: A Retrospective Study From Turkey Between 1996 and 2023

**DOI:** 10.1111/cod.70000

**Published:** 2025-07-12

**Authors:** İbrahim Halil Aydoğdu, Esen Özkaya

**Affiliations:** ^1^ Department of Dermatology and Venereology, İstanbul Faculty of Medicine İstanbul University İstanbul Turkey

**Keywords:** allergic contact dermatitis, carbamate, epidemiology, glove, mercapto, nonoccupational, occupational, patch test, rubber additives, thiuram

## Abstract

**Background:**

Rubber additives are common causes of allergic contact dermatitis (ACD) worldwide, yet data from Turkey remain limited.

**Objectives:**

To evaluate the prevalence of rubber additive sensitisation and its clinical/occupational relevance in a tertiary referral centre.

**Methods:**

A retrospective study on 2687 consecutively patch‐tested patients with rubber additives at our allergy unit between 1996 and 2023.

**Results:**

Rubber sensitisation was found in 10.6%, with a slight increase after 2010. Male predominance was noted (female: male = 1:2.3). Thiurams and carbamates were the most frequent sensitizers. ACD was diagnosed in 7.9%, primarily caused by gloves (85.5%). Hands were most commonly affected (94.4%). Logistic regression analysis showed that thiuram and carbamate sensitisation was significantly associated with hand eczema, while benzothiazole derivatives were linked to foot eczema. Airborne ACD occurred in 4.2%, mainly in healthcare workers. Hand eczema with and without wrist extension was observed with similar frequency. Occupational ACD accounted for 79.3% of cases, especially among construction (56.8%) and healthcare workers (14.8%), with a relative increase in the latter group after 2015.

**Conclusions:**

The high prevalence of sensitisation from rubber gloves is concerning. Legal measures are urgently needed, including safer additives and clearer glove labelling. Patch testing remains essential, even without the classic glove‐pattern distribution.

## Graphical abstract

The overall prevalence of sensitisation to rubber additives between 1996 and 2023 was 10.6%, with thiurams and carbamates being the most common sensitisers. Allergic contact dermatitis (ACD) was diagnosed in 7.9% (213/2687) of patients predominantly due to glove use. Airborne ACD was diagnosed in 4.2%, mainly among healthcare workers. Thiurams and carbamates were significantly associated with hand eczema, while benzothiazole derivatives were linked to foot eczema. Most rubber‐induced ACD cases were occupational, particularly among construction and healthcare workers. While occupational ACD declined over the past decade, nonoccupational ACD showed a relative increase.

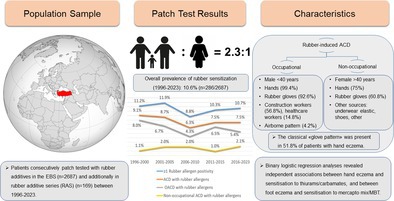

## 1 Introduction

Rubber additives such as thiurams are well‐recognised culprits of rubber‐induced allergic contact dermatitis (ACD) worldwide. The most commonly implicated products in sensitisation to rubber additives include gloves, shoes and laundry elastics [1, 2, 3, 4]. Rubber‐induced occupational ACD (OACD) is particularly common, especially among construction workers and healthcare professionals, with gloves serving as a major source [5].

The aim of this study was to evaluate the prevalence of contact sensitisation to rubber additives and examine their clinical and occupational relevance in a relatively large patient series from a tertiary referral centre in Turkey, a country with limited data on this subject.

## 2 Methods/Materials

This tertiary, monocentre retrospective study analysed the files of 2687 patients consecutively patch tested with our extended European baseline series, including rubber additives, for clinically suspected ACD of any origin and/or rubber allergy at the allergy unit of our clinic between June 1996 and March 2023 (Table 1). A small proportion of patients were additionally tested with a rubber additives series (RAS), where indicated.

**TABLE 1:** Patch test results with rubber additives in our extended European baseline series and rubber series at our department between 1996 and 2023.

Extended baseline series rubber allergens	Positive/tested patients *n* (%)
Thiuram mix 1% pet.	160/2683 (6.0)
Mercapto mix 2% pet.	30/2675 (1.1)
MBT 2% pet.	42/2682 (1.6)
Carba mix 3% pet.^a^	71/1521 (4.7)
ZDEC 1% pet.^b^	39/1190 (3.3)
N‐isopropyl‐N‐phenyl‐4‐phenylenediamine 0.1% pet.	36/2649 (0.1)
4,4′‐Diaminodiphenylmethane 0.5% pet.	44/2649 (0.5)
Mercapto mix and/or MBT	46/2683 (1.7)
Carba mix and/or ZDEC	110/2648 (4.2)

Rubber series	
Tetramethylthiuram disulfide 1% pet.	17/152 (11.2)
Tetramethylthiuram monosulfide 1% pet.	20/152 (13.2)
Dipentamethylene thiuram disulfide 1% pet.	4/152 (2.6)
Tetraethylthiuram disulfide 1% pet.	26/152 (17.1)
ZDEC 1% pet.	9/144 (6.3)
Zinc dibutyldithiocarbamate 1% pet.	4/149 (2.7)
1,3‐Diphenylguanidine 1% pet.	13/150 (8.7)
N‐Cyclohexyl‐2‐benzothiazyl sulfonamide 1% pet.	5/151 (3.3)
Dibenzothiazyl disulfide 1% pet.	4/142 (2.8)
2‐(4‐Morpholinylmercapto) benzothiazole 1% pet.	5/141 (3.5)
MBT 2% pet.	13/169 (7.7)
4,4′‐Diaminodiphenylmethane 0.5% pet.	3/154 (1.9)
N,N‐Diphenyl‐p‐phenylenediamine 1% pet.	0/149 (0)
N‐Cyclohexyl‐N‐phenyl‐4‐phenylenediamine 1% pet.	0/118 (0)
Ethylenediamine dihydrochloride 1% pet.	0/151 (0)
N,N‐Di‐2‐naphtyl‐p‐phenylenediamine 1% pet.	0/111 (0)
N‐Phenyl‐2‐naphtylamine 1% pet.	0/147 (0)
N,N′‐Diphenylthiourea 1% pet.	0/156 (0)
N,N′‐Dibutylthiourea 1% pet.	0/156 (0)
N,N′‐Diethylthiourea 1% pet.	1/126 (0.8)
Hydroquinone monobenzylether (monobenzone) 1% pet.	0/143 (0)
Methenamine 2% pet.	0/149 (0)
Benzoylperoxide 1% pet.	2/152 (1.3)
Tricresyl phosphate 5% pet.	0/151 (0)
N‐(Cyclohexylthio) phthalimide 1% pet.	5/152 (3.3)
2,2,4‐Trimethyl‐1,2‐dihydroquinoline 1% pet.	1/128 (0.8)

Abbreviations: MBT, mercaptobenzothiazole; ZDEC, zinc diethyldithiocarbamate.

^a^ In our extended European baseline series, carba mix was tested without zinc diethyldithiocarbamate between 1996 and 2009, and with zinc diethyldithiocarbamate since 2021.

^b^ In our extended European baseline series, zinc diethyldithiocarbamate was tested without carba mix between 2010 and 2020 and with carba mix since 2021.

Test allergens were provided by Chemotechnique Diagnostics (Vellinge, Sweden), Brial Allergen (Greven, Germany), AllergEAZE (Calgary, Canada) and earlier by Hal‐Brial (Leiden, The Netherlands). Patch testing was conducted following verbal or written consent from patients or their legal guardians. Patients refrained from using antihistamines, nonsteroidal anti‐inflammatory drugs or immunosuppressive agents, topical or systemic corticosteroids for at least 1 month before and during patch testing. Pregnant or lactating women were not tested.

These allergens were applied using IQ Chambers (Chemotechnique Diagnostics) on the upper back for 48 h, occluded with Hypafix tape (BSN medical GmbH, Hamburg, Germany). Patients' own rubber products (e.g., gloves, shoes, thermal socks, rubber mats, elastic bands, masks, hoses, gear knobs, steering wheel covers) were patch tested for 72 h (pieces of 2 × 2 cm^2^ or larger, moistened with water). Readings were performed on days (D) 2, 3, 4 and, from 2010 onward, also on D7, following the European Society of Contact Dermatitis (ESCD) patch test guidelines [6]. Patch test methodology and technical procedures have remained consistent in our clinic since 1996. Positive patch test reactions were recognised only if they exhibited crescendo or plateau‐positive patterns on at least 2 consecutive days.

Patient files were reviewed for demographic features (age, sex, presence of atopic dermatitis according to Hanifin and Rajka criteria [7] and atopic skin diathesis according to Erlangen atopic score criteria [8]), patch test results, frequency and progression of positive reactions over the years, clinical/occupational relevance and sources of rubber contact. Standardised forms were used for precise documentation of occupational and nonoccupational ACD.

The MOAHLFA index (male sex, occupational dermatitis, atopic dermatitis/atopic skin diathesis, hand dermatitis, leg dermatitis, face dermatitis, age > 40 years) [9] including foot dermatitis [10] was calculated, and compared between patients with and without rubber additive sensitisation. Clinical relevance was determined based on history and clinical findings confirming anatomical and temporal relationship with exposure, supplemented by additional patch testing with rubber products, when appropriate. Rubber‐induced airborne ACD was diagnosed if eczema lesions were located on air‐exposed areas, there was exposure to airborne rubber particles, particularly from frequent use of powdered latex gloves by the patient or those in close proximity, patch testing was positive exclusively to rubber additives, and clinical and occupational relevance could be established.

The COADEX system was used to assign relevance to positive patch test reactions, using the following categories: C (current)—O (old)—A (active sensitisation)—D (doubtful)—E (exposed)—X (cross‐reaction) [11]. Occupational relevance was assessed according to Mathias criteria [12].

Statistical analyses were performed using SPSS version 26 (IBM, Armonk, New York). Data were evaluated using descriptive statistics (mean, standard deviation, median, frequency, percentage, minimum, maximum). Pearson chi‐square tests compared categorical data. Binary logistic regression analysis was used to evaluate the independent associations between MOAHLFA criteria and ACD from rubber additives, specific rubber allergens (thiuram mix, carba mix/zinc diethyldithiocarbamate [ZDEC] and mercapto mix/mercaptobenzothiazole [MBT]). Statistical significance was set at *p* < 0.05.

This study was approved by the ethics committee of Istanbul University, Istanbul Faculty of Medicine (approval number: 2023/690), and conducted in accordance with the Declaration of Helsinki.

## 3 Results

Patch testing was conducted with baseline series rubber allergens in 2687 patients, and with an additional RAS on 169 patients (Table 1). Among these, 10.6% (286/2687) showed a positive patch test reaction to at least one rubber allergen. The characteristic features of these patients are presented in Table 2.

**TABLE 2:** Characteristics of patients with positive patch test reactions to rubber additives.

Patients' characteristics	Patients with positive patch test reaction to rubber allergens (*n* = 286)
Gender, male:female, *n* (ratio)	86:200 (1:2.3)
Age, range (mean), years	3–79 (38.9)
Female	8–68 (35.3)
Male	3–79 (40.4)
Atopic dermatitis/atopic skin diathesis, *n* (%)	18 (6.3)
Overall prevalence of sensitisation to ≥ 1 rubber allergen	10.6% (286/2687)
Clinical relevance, *n* (%)	7.9% (213/2687)
Localisation of rubber‐induced ACD (*n* = 213)	Hands (*n* = 201/213, 94.4%) comprising hands only (*n* = 164), hand and airborne (*n* = 9/213, 4.2%), hand and generalised (*n* = 10), hand and foot (*n* = 3), hand and other (*n* = 5) Rubber contact sites excluding gloves (*n* = 7) Foot (*n* = 5)
Sources of rubber‐induced ACD (*n* = 227)^a^	Gloves (*n* = 194/227, 85.5%), shoes (*n* = 9/227, 4%), underwear elastic (9/227, 4%), face mask (*n* = 3/227, 1.3%),^b^ other (12/227, 5.3%)^b^
Occupational ACD	79.3% (169/213)
Gender, male:female, *n* (ratio)	144:25 (5.8:1)
Age, range (median), years	17–79 (38.6)
Age > 40 years, *n* (%)	65 (38.5)
Duration of disease, range (median), months	0.3–360 (82.2)
Hand involvement, *n* (%)	168/169 (99.4)
Airborne pattern, *n* (%)	9/169 (5.3)
Occupations	Construction workers (*n* = 96/169, 56.8%), health care workers (*n* = 25/169, 14.8%), metal workers (*n* = 8), cleaning workers (*n* = 6), hairdressers (*n* = 4), rubber factory worker (*n* = 1), others (29)
Sources of rubber‐induced occupational ACD (*n* = 176)^a^	Gloves (*n* = 163/176, 92.6%), shoes (*n* = 3), face mask (*n* = 3), other (*n* = 7)
Nonoccupational relevance	20.7% (44/213)
Gender, male:female, *n* (ratio)	17:27 (1:1.6)
Age, range (median), years	3–77 (41)
Age > 40 years, *n* (%)	25 (56.8)
Duration of disease, range (median), months	0.5–540 (86.4)
Hand involvement, *n* (%)	33/44 (75)
Sources of rubber‐induced nonoccupational ACD (*n* = 51)^a^	Gloves (*n* = 31/51, 60.8%), underwear elastic (*n* = 9), shoes (*n* = 6), other (*n* = 5)

Abbreviation: ACD, allergic contact dermatitis.

^a^ The total number of sources exceeded the number of patients, as some patients had more than one source of rubber sensitisation.

^b^ Agricultural pesticides, neoprene thermal socks, black cables, rubber bands, steering wheel covers, gear knobs, industrial felt, knee braces, bicycle handlebar covers, basketballs and rubber hose.

**FIGURE 1:**
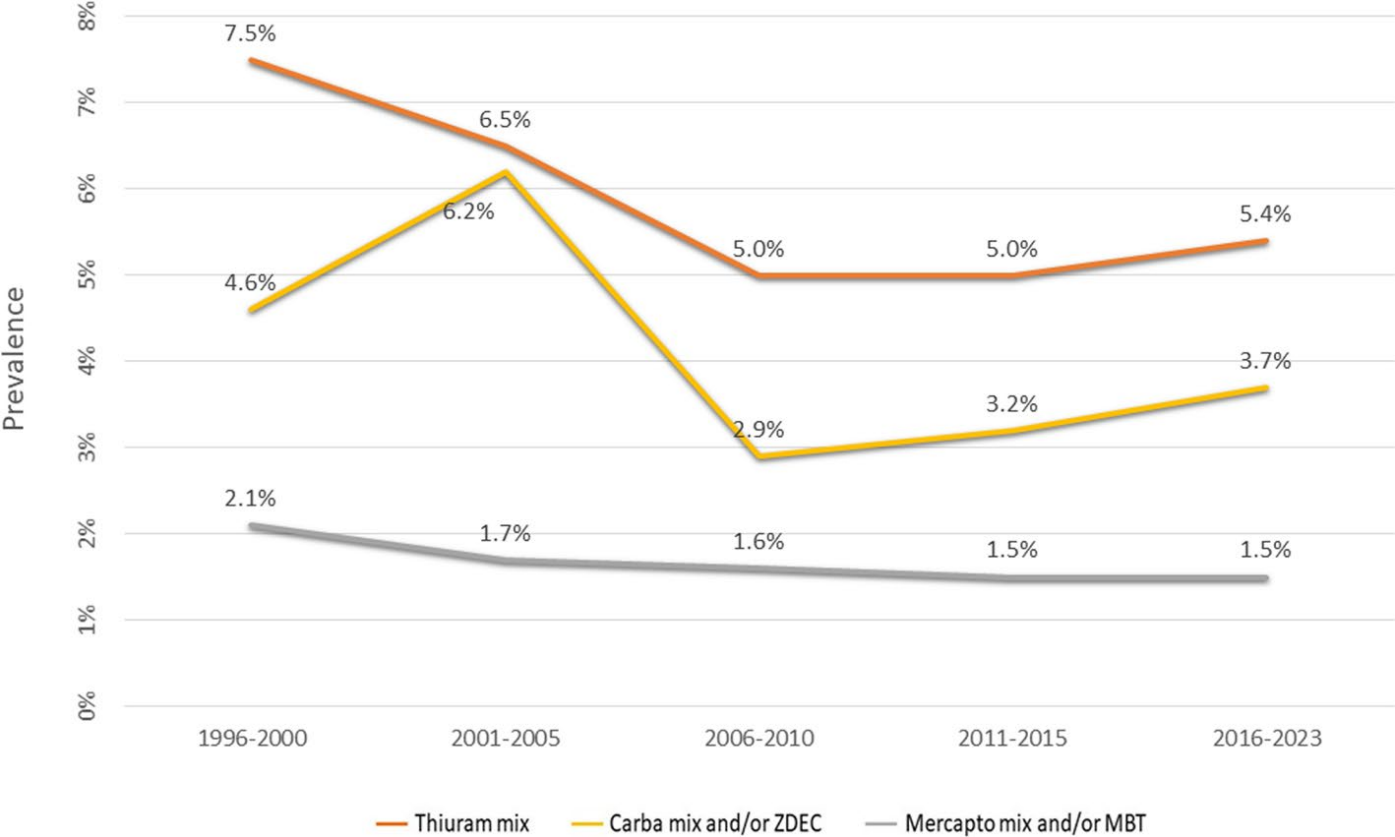
Temporal changes in the prevalence of baseline series rubber allergens.

Patch test positivity to rubber additives was significantly associated with male sex (female: male = 1:2.3) (*p* < 0.001) (Table 3). The most frequent sensitisers in our extended European baseline series were thiuram mix (6%), and carba mix and/or ZDEC (4.2%) (Table 1). Among the 286 patients with a positive patch test reaction to at least one rubber allergen, 78 (27.3%) showed reactions to both thiurams and dithiocarbamates. While the positivity rates for thiuram mix and carba mix and/or ZDEC declined between 1996 and 2010, an increase was observed from 2010 onward (Figure 1). Sensitisation to mercapto mix and/or MBT (1.7%) remained relatively stable throughout the study period.

**TABLE 3:** Comparison of MOAHLFA parameters, including foot dermatitis, between patients with and without rubber sensitisation in our study from 1996 to 2023. Significant *p* values are highlighted in bold.

MOAHLFA parameters, including foot dermatitis	Patients with rubber sensitisation *n* = 286 *n* (%)	Patients without rubber sensitisation *n* = 2401 *n* (%)	*p* ^a^
Male sex	200 (69.9)	1039 (43.3)	**< 0.001** (OR = 3.1, 95% CI: 2.3–4.0)
Occupational dermatitis	169 (59.1)	188 (7.8)	**< 0.001** (OR = 17.0, 95% CI: 12.9–22.5)
Atopic dermatitis/atopic skin diathesis	18 (6.3)	187 (7.8)	0.37
Hand dermatitis	234 (81.8)	1376 (57.3)	**< 0.001** (OR = 3.4, 95% CI: 2.5–4.6)
Leg dermatitis	34 (11.9)	335 (14.0)	0.34
Face dermatitis	75 (26.2)	674 (28.1)	0.51
Age > 40 years	126 (44.1)	941 (39.5)	0.14
Foot dermatitis	56 (19.6)	409 (17.0)	0.28

Abbreviations: CI, confidence interval; OR, odds ratio.

^a^ *p* values were calculated using the Chi‐square test.

Additional positive patch test reactions to nonrubber contact allergens such as chromium, cobalt, nickel, palladium, thimerosal, fragrance mix, *Myroxylon pereirae* , methylchloroisothiazolinone/methylisothiazolinone (MCI/MI) and MI were observed in 81.5% (233/286) of patients sensitised to rubber allergens. Concomitant contact sensitisation to chromate and rubber allergens was seen in 37.8% (108/286) (*p* < 0.001; odds ratio [OR]: 10.1; 95% confidence interval [CI]: 7.5–13.6).

**FIGURE 2:**
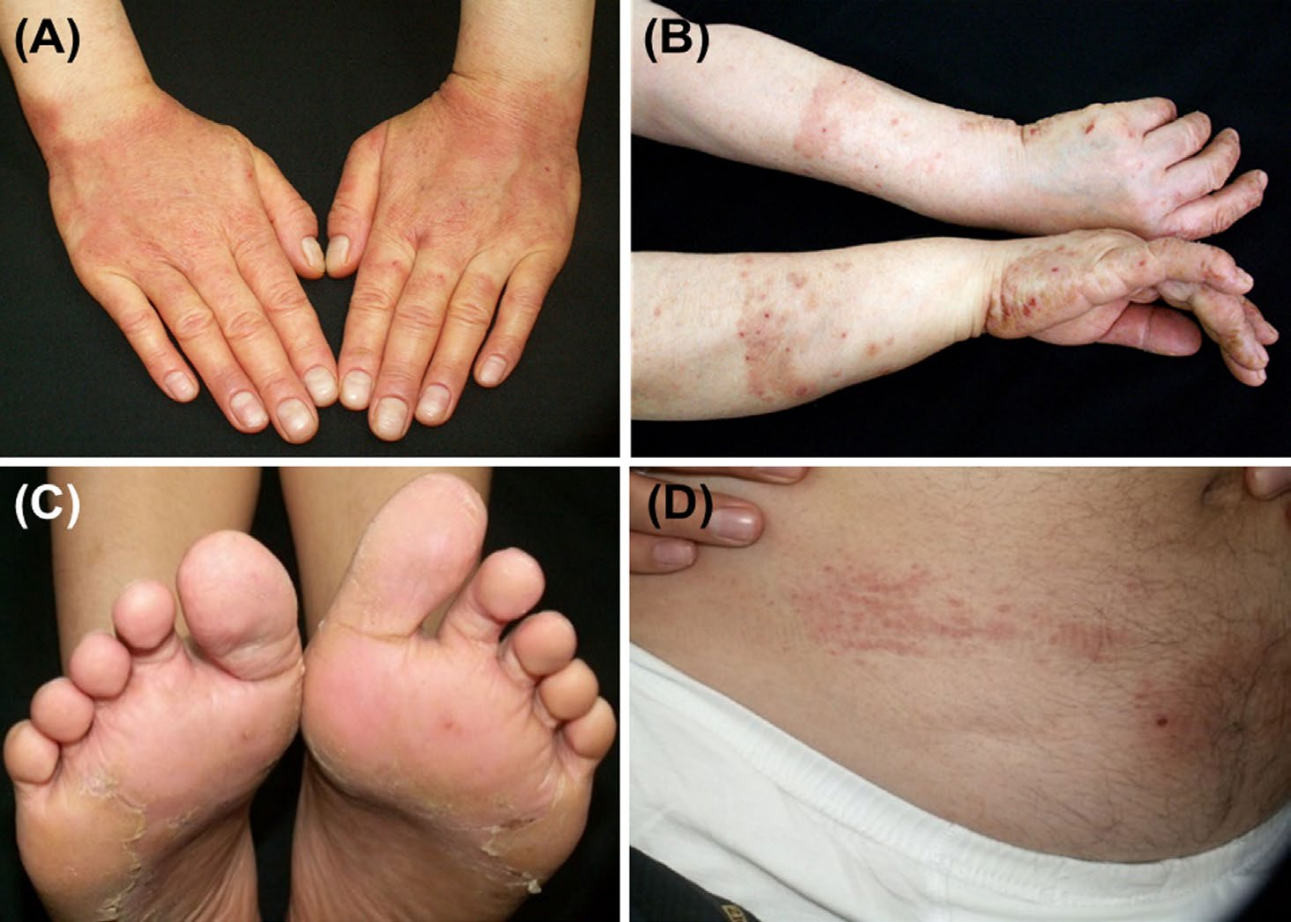
Rubber‐induced allergic contact dermatitis from (A) work gloves, with involvement of the wrists, (B) household gloves, showing the typical glove pattern on the forearms, (C) footwear, affecting the soles and (D) underwear elastic, corresponding to the contact area on the body.

**FIGURE 3:**
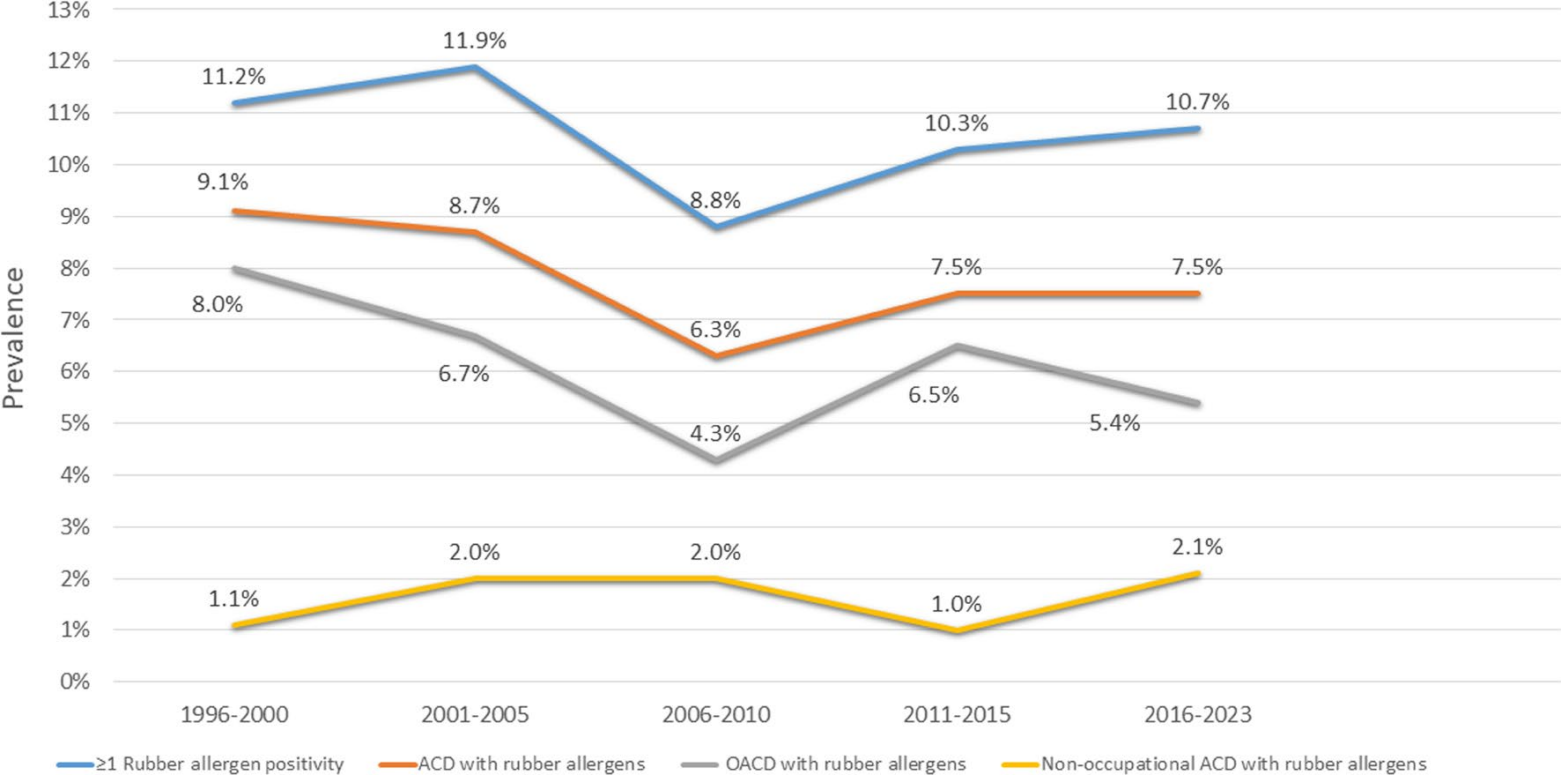
Temporal changes in the prevalence of rubber allergen positivity, rubber‐induced allergic contact dermatitis (ACD), occupational ACD (OACD) and nonoccupational ACD over a 28‐year study period.

Rubber‐induced ACD was diagnosed in 7.9% (213/2687) of patients, with the hands being the most frequently affected site (94.4%) (Table 2). A characteristic ‘glove pattern’ (Figure 2), with hand eczema extending beyond the wrists or forearms, was seen in 51.8% of patients. Airborne ACD was diagnosed in nine out of 213 patients (4.2%) with rubber‐induced ACD, all of whom had OACD; specifically, five nurses, two medical doctors and two factory workers. The prevalence of rubber‐induced ACD decreased between 1996 and 2010 but slightly increased after 2010 (Figure 3).

Rubber‐induced ACD was diagnosed with baseline series rubber allergens in 209/213 (98.1%), whereas ACD could not be detected in 4/213 (1.9%) patients without patch testing with the RAS. The culprit rubber allergens in these four patients were N‐(cyclohexylthio) phthalimide 1% pet. (*n* = 2), N, N'‐diethylthiourea1% pet. (*n* = 1), and 2,2,4‐Trimethyl‐1,2‐dihydroquinoline 1% pet. (*n* = 1). The number of patients whose diagnosis of rubber‐induced ACD was based solely on positive patch test reactions to thiuram mix, carba mix and mercapto mix was 4, 6 and 4, respectively, without any supportive reactions to the individual constituents of these mixes.

A total of 227 rubber‐containing products were responsible for rubber‐induced ACD, primarily including gloves (85.5%), shoes (4%) and laundry elastics (4%) (Table 2). Among the gloves implicated, nitrile (101/194, 52.1%) and latex gloves (36/194, 18.6%) were most commonly associated with rubber‐induced ACD.

**FIGURE 4:**
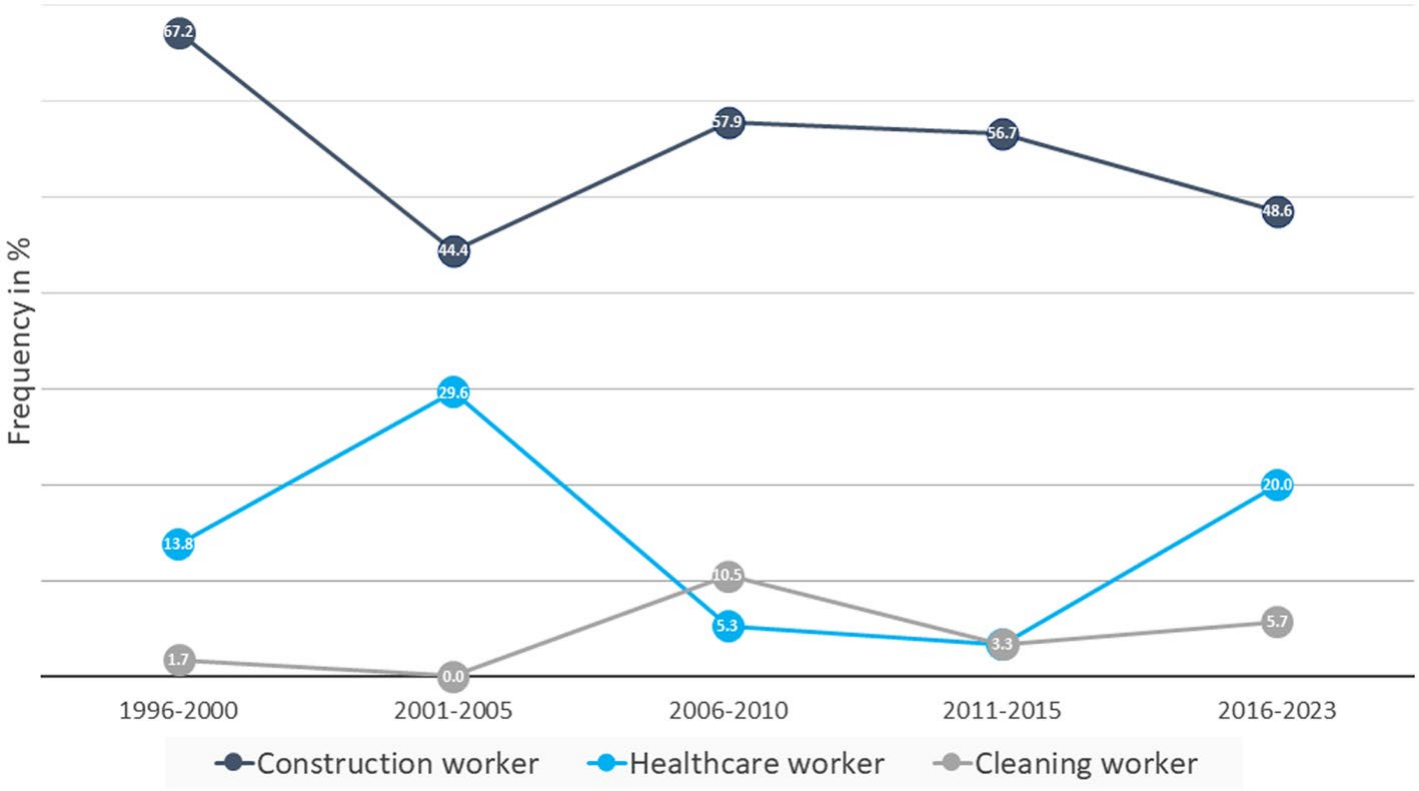
Temporal changes in the frequency of rubber‐induced OACD according to occupations.

Rubber‐induced ACD was occupational in 79.3% of patients (169/213). OACD was notably prevalent among construction workers and healthcare workers (Table 2), with a relative increase in the latter group observed after 2015 (Figure 4). Gloves, shoes and medical masks were the most frequent sources of rubber‐induced OACD (Table 2).

Nonoccupational rubber‐induced ACD (44/213, 20.7%) also predominantly affected the hands (75%), primarily due to gloves, whereas underwear and shoes were other relevant sources.

Male sex and age ≤ 40 years were more prominent in cases of rubber‐induced ACD and OACD, whereas female sex and age > 40 years were more common in nonoccupational rubber‐induced ACD (Table 2). Binary logistic regression analysis identified occupation, rather than sex, as the primary determinant of rubber‐induced ACD (Table 4). Additionally, benzothiazole derivatives were significantly associated with foot eczema, whereas thiurams and carbamates were more strongly linked to hand eczema (Table 4).

**TABLE 4:** Logistic regression analysis of MOAHLFA parameters, including foot dermatitis, in patients with rubber‐induced allergic contact dermatitis (ACD), and confirmed ACD from thiuram mix, carba mix/zinc diethyldithiocarbamate and mercapto mix/mercaptobenzothiazole. Significant odds ratio values and 95% confidence intervals are highlighted in bold.

MOAHLFA parameters, including foot dermatitis	1. Patients with ACD from rubber additives (*n* = 213) *n* (%)	2. Patients without ACD from rubber additives (*n* = 2474) *n* (%)	2 versus 1 adjusted OR (95% CI)	3. Patients with ACD from thiuram mix (*n* = 150) *n* (%)	4. Patients without ACD from thiuram mix (*n* = 2537) *n* (%)	4 versus 3 adjusted OR (95% CI)	5. Patients with ACD from carba mix and/or ZDEC (*n* = 93) *n* (%)	6. Patients without ACD from carba mix and/or ZDEC (*n* = 2594) *n* (%)	6 versus 5 adjusted OR (95% CI)	7. Patients with ACD from mercapto mix and/or MBT (*n* = 38) *n* (%)	8. Patients without ACD from mercapto mix and/or MBT (*n* = 2649) *n* (%)	8 versus 7 adjusted OR (95% CI)
Male sex	161 (75.6)	1078 (43.6)	1.1 (0.7–1.7)	111 (74.0)	1128 (44.5)	0.8 (0.5–1.3)	70 (75.3)	1169 (45.1)	1.0 (0.5–1.7)	31 (81.6)	1208 (45.6)	1.3 (0.5–3.2)
Occupational dermatitis	169 (79.3)	188 (7.6)	**36.0 (23.7–54.7)**	126 (84.0)	231 (9.1)	**36.0 (21.7–59.8)**	78 (83.9)	279 (10.8)	**36.9 (19.2–70.7)**	32 (84.2)	325 (12.3)	**33.0 (11.7–95.2)**
Atopic dermatitis/atopic skin diathesis	13 (6.1)	192 (7.8)	1.5 (0.7–3.0)	9 (6.0)	196 (7.7)	1.3 (0.6–3.1)	7 (7.5)	198 (7.6)	1.9 (0.8–4.7)	1 (2.6)	204 (7.7)	0.6 (0.1–4.5)
Hand dermatitis	201 (94.4)	1409 (57.0)	**3.5 (1.8–6.9)**	148 (98.7)	1462 (57.6)	**16.4 (3.8–69.7)**	87 (93.5)	1523 (58.7)	**2.7 (1.04–7.1)**	35 (92.1)	1575 (59.5)	1.1 (0.3–4.3)
Leg dermatitis	18 (8.5)	351 (14.2)	**0.5 (0.3–0.97)**	10 (6.7)	359 (14.2)	**0.5 (0.2–0.95)**	8 (8.6)	361 (13.9)	0.6 (0.3–1.4)	1 (2.6)	368 (13.9)	0.1 (0.0–1.1)
Face dermatitis	49 (23.0)	700 (28.3)	1.1 (0.7–1.7)	35 (23.3)	714 (28.1)	1.2 (0.8–2.0)	27 (29.0)	722 (27.8)	1.5 (0.9–2.5)	6 (15.8)	743 (28.0)	0.7 (0.3–1.7)
Age > 40 years	90 (42.3)	977 (39.8)	**2.1 (1.4–3.0)**	59 (39.3)	1008 (40.0)	**1.8 (1.2–2.7)**	45 (48.4)	1022 (39.7)	**2.5 (1.5–4.0)**	16 (42.1)	1051 (39.9)	1.4 (0.7–2.9)
Foot dermatitis	48 (22.5)	417 (16.9)	1.3 (0.8–2.0)	29 (19.3)	436 (17.2)	0.9 (0.6–1.6)	19 (20.4)	446 (17.2)	1.0 (0.6–1.8)	14 (36.8)	451 (17.0)	**2.9 (1.4–6.1)**

Abbreviations: ACD, allergic contact dermatitis; CI, confidence interval; MBT, mercaptobenzothiazole; OR, odds ratio; ZDEC, zinc diethyldithiocarbamate.

## 4 Discussion

### 4.1 Demographic Features

This study presents the largest dataset on patch test results with rubber allergens from Turkey over a 28‐year period. A significant association was observed between male gender and rubber allergen positivity, ACD and OACD, likely due to the high prevalence of occupational sensitisation in construction work, a key profession linked to rubber allergen‐induced OACD in Turkey [13]. In contrast, a Danish study found no gender differences, possibly reflecting the gender‐balanced cohort of healthcare workers [14]. Bendewald et al. reported female predominance among rubber‐sensitised patients in the United States, though occupational factors were unspecified [15]. Multivariable analyses in our study confirmed that occupation, rather than gender, was the primary determinant of rubber‐induced ACD. This finding was in accordance with research from Taiwan, where construction workers were similarly most affected [16].

The link between age and rubber sensitisation remains inconsistent in the literature. Our findings were similar to previous studies reporting increased sensitisation with age [10, 17], likely reflecting the high number of patients with OACD, particularly among construction workers. Atopic dermatitis or atopic skin were not associated with rubber allergen sensitisation in our series, aligning with prior studies [14, 16, 18].

### 4.2 Rubber Additives

The prevalence of sensitisation to rubber additives in our study was generally higher than in most other countries (Table 5) [14, 15, 16, 19, 20, 21]. This elevated prevalence in our patient group likely reflects the high proportion of OACD cases with the predominance of construction workers, who were frequently sensitised to rubber gloves. Bendewald et al. reported the highest prevalence (31.7%), possibly due to counting macular erythema and doubtful reactions as positive [15].

**TABLE 5:** Comparison of the frequency of rubber sensitivity in the present and the previously published studies.

Author (year)	Country	Total number of tested patients, *n*	At least one rubber allergen positivity, *n* (%)
Aydoğdu İH & Özkaya E (present study)	Turkey	2687	286 (10.6)
Kursawe Larsen et al. (2024) [14]	Denmark	24 373	652 (2.7)
Chen et al. (2004) [16]	Taiwan	2235	99 (4.4)
Conde‐Salazar et al. (1993) [20]	Spain	4680	686 (14.7)
Von Hintzenstern et al. (1991) [19]	Germany	3851	145 (3.8)
Bendewald et al. (2010) [15]	USA	739^a^	245 (31.7)
Holness et al. (1997) [21]	USA	1670	149 (8.9)

Abbreviation: USA, United States of America.

^a^ Inclusion of macular erythema in the list of positive reactions.

Unfortunately, we were unable to patch test all patients who showed a rubber allergen reaction in the baseline series with the RAS subsequently, primarily due to patients' reluctance to undergo a second round of patch testing. Preferably, one should patch test with a RAS in suspected rubber contact allergy and in patients who show a positive reaction to a rubber mix in the baseline series.

In our study, thiuram mix showed the highest positivity (6%), followed by carba mix and/or ZDEC (4.2%), consistent with most previous studies that identified thiuram mix as the most frequently positive allergen [16, 19, 20, 21, 22]. This predominance may reflect their historical use as cost‐effective rubber accelerators, the superior diagnostic capability of thiurams for thiuram/dithiocarbamate redox pairs in patch testing, or structural similarities with carbamates [23, 24]. It is to be noted that both false‐positive and false‐negative reactions to thiuram mix have been reported, and that positive reactions to dithiocarbamates without concomitant thiuram allergy are rare [25]. In our patient group, thiuram mix and carba mix/ZDEC remained the most frequently identified allergens throughout the study period, with a resurgence in positivity after 2010, the latter likely resulting from the increased use of carbamates in rubber gloves in recent years [26, 27].

A decline in thiuram sensitisation was observed in the late 1990s [17, 26, 28, 29], coinciding with the growing use of carbamates, benzothiazoles and 1,3‐diphenylguanidine (DPG) in rubber products, particularly medical gloves, and the reduced use of thiurams in glove manufacturing [5, 26, 30]. Warburton et al. noted an increasing trend in carba mix positivity between 1996 and 2012 [31], consistent with ESSCA data from 2004 to 2012 [10], and other studies [32, 33], possibly due to the rising use of carbamate accelerators in rubber gloves [26, 27]. On the other hand, carba mix is more prone to causing irritant reactions due to 1,3‐DPG, one of its components, which may lead to false‐positive results [26, 34], and potentially contribute to higher reported sensitisation rates to carba mix in some cohorts.

### 4.3 Rubber‐Induced ACD

Rubber‐induced ACD was primarily caused by gloves, as shown in both the present and previous studies [14, 19], across occupational and nonoccupational settings. In our study, logistic regression analysis identified the hands as a site significantly associated with thiuram mix positivity, consistent with the findings by Kursawe Larsen et al. [14] and others [10, 16, 17, 35]. Multivariable analyses further revealed significant associations between hand eczema and both thiuram mix and carba mix/ZDEC, the historical and current rubber glove ingredients, respectively.

In glove‐associated ACD, eczema typically appears on the dorsum of the hand and extends beyond the wrist, following the outline of the gloves [36]. In our study, the frequency of hand involvement with and without wrist extension was nearly equal, underscoring the importance of patch testing for a definitive diagnosis of rubber glove allergy, even in the absence of the typical glove pattern.

For patients sensitised to rubber additives, accelerator‐free gloves are recommended [37]. However, some gloves labelled as accelerator‐free have been shown to contain accelerators [38, 39, 40, 41]. Polyethylene glove liners are recommended for protection against glove allergens of the actual protective glove [42, 43].

In our study, foot eczema was significantly associated with mercapto mix/MBT, in accordance with studies identifying thiazoles and MBT as primary causes of footwear‐related dermatitis [14, 44]. ESSCA data reported a higher prevalence of foot eczema among MBT‐positive patients, particularly in younger individuals [10, 14, 18, 35]. Chen et al. highlighted mercapto mix as a major cause of foot eczema in Taiwan, likely due to rubber sandals and increased allergen release from sweating in hot weather [16]. In rubber‐induced foot eczema, the plantar surface of the feet is mostly affected, likely associated with rubber‐based adhesives in shoe soles and linings [1, 36]. Recently, a rare case of ACD was reported from diethylthiourea and carbamates in neoprene prayer socks [45].

Patients with persistent dermatitis despite avoiding allergenic shoes should be informed that allergens may remain in socks, even after washing at high temperatures [46]. For prevention, they should choose shoes with insoles made from nonrubber materials, such as cork, composite or felt and adhesives free of rubber derivatives. Barrier socks, creams and treatment for hyperhidrosis, if present, are also recommended [47].

Rubber‐induced ACD was also associated with underwear elastics in our series. ACD caused by rubber additives should be considered in cases of eczema at the site of underwear or sock elastics [4]. Rubber‐based elastomers are still used in some underwear, although less commonly than modern textile elastomers, which are typically made from elastane, a polyurethane‐based synthetic polymer. Additionally, rubber additives in mask straps can contribute to mask‐associated ACD [48], which is common among healthcare workers [49], as observed during the COVID‐19 pandemic.

### 4.4 Rubber‐Induced OACD

In our patient group, rubber‐induced OACD was most common among construction and healthcare workers, occupations with prolonged glove exposure and wet work [14, 32, 50]. High rates of coexisting chromium sensitisation in construction workers with rubber allergy, as observed in the present and the previous studies [16, 19, 20], suggest that the irritant effect of cement, due to its alkalinity, along with microfrictions, may promote sensitisation by impairing the epidermal barrier. A previous study from our clinic also highlighted a strong association between thiuram and chromium allergies, almost serving as a marker for construction workers [13]. Based on this finding, the decline in rubber‐induced OACD among construction workers in the present study was likely linked to the decrease in chromium‐induced OACD in this population [13], which began even before the implementation of the national cement regulation in Turkey, probably due to the increased use of precast concrete blocks instead of bagged cement in the construction industry.

The reduction in glove‐related rubber sensitisation among construction workers after cement regulation resulted in a relative increase in OACD among healthcare workers in our study. Similarly, in Europe and the USA, rubber‐induced OACD is most frequently observed among healthcare workers [10, 14, 15, 17, 51].

Hairdressers were not significantly affected, likely due to infrequent glove use or the preference for cheaper polyethylene gloves [13, 52]. A notable group of patients with rubber‐induced ACD were housewives in our study. ACD caused by household gloves is less common than that caused by work gloves, likely due to factors such as cotton linings, intermittent use [30], and the lower release of thiurams and carbamates from household gloves compared to surgical gloves [53].

In contrast to the reduction in OACD, nonoccupational ACD increased in our cohort, aligning with findings by Warburton et al. regarding a similar trend [31]. This highlights the need for additional measures to address nonoccupational sources of rubber allergens.

A notable proportion of our patients had rubber‐induced airborne OACD, particularly healthcare workers. These patients were sensitised exclusively to rubber additives and had significant exposure to airborne rubber allergens in the workplace, either through frequent glove changes or proximity to colleagues doing so. Rubber‐induced airborne ACD is rarely reported in the literature [13, 54, 55, 56], and is likely underreported, probably due to a lack of awareness of this condition, diagnostic challenges, limited use of patch testing or the infrequent publication of such cases [57, 58].

### 4.5 Limitations and Strengths

The primary limitations of this study were its retrospective design and the fact that only a small minority of the patients were patch tested with the RAS. However, its strengths include a large patient group, a long study period, and the use of standardised documentation forms. Importantly, only patients who underwent a systematic and comprehensive assessment of clinical and occupational relevance were included, which represents a major strength of the study.

## 5 Conclusion

The persistently high sensitisation rates to thiurams in gloves, without a significant decrease over time, are concerning. Thiurams and carbamates were strongly associated with hand eczema, while benzothiazole derivatives were predominantly linked to foot eczema. Urgent legal measures are needed, both in terms of production (e.g., reducing the use of highly sensitising rubber additives) and consumer safety (e.g., clear labelling of glove composition), particularly to protect construction workers and healthcare professionals. Finally, the considerable number of patients with rubber‐induced ACD from gloves who lacked the classical glove‐pattern distribution, as well as those presenting with airborne ACD, highlights the importance of patch testing for accurate diagnosis and points to the underreported nature of rubber‐related airborne ACD.

## Author Contributions

**İbrahim Halil Aydoğdu:** conceptualization, methodology, data curation, investigation, validation, formal analysis, visualization, writing – original draft. **Esen Özkaya:** conceptualization, methodology, data curation, investigation, validation, formal analysis, supervision, visualization, writing – review and editing.

## Conflicts of Interest

The authors declare no conflicts of interest.

## Data Availability

Research data are not shared.
